# Improved power and long term performance of microbial fuel cell with Fe-N-C catalyst in air-breathing cathode

**DOI:** 10.1016/j.energy.2017.11.135

**Published:** 2018-02-01

**Authors:** Iwona Gajda, John Greenman, Carlo Santoro, Alexey Serov, Chris Melhuish, Plamen Atanassov, Ioannis A. Ieropoulos

**Affiliations:** a Bristol BioEnergy Centre, Bristol Robotics Laboratory, University of the West of England, BS16 1QY, UK; b Biological, Biomedical and Analytical Sciences, University of the West of England, BS16 1QY, UK; c Center for Micro-Engineered Materials (CMEM), Department of Chemical and Biological Engineering, University of New Mexico, Albuquerque, NM, USA

**Keywords:** Microbial fuel cell, Cathode catalyst, Catholyte extraction, Caustic catholyte, Electro-osmosis

## Abstract

Power output limitation is one of the main challenges that needs to be addressed for full-scale applications of the Microbial Fuel Cell (MFC) technology. Previous studies have examined electrochemical performance of different cathode electrodes including the development of novel iron based electrocatalysts, however the long-term investigation into continuously operating systems is rare. This work aims to study the application of platinum group metals-free (PGM-free) catalysts integrated into an air-breathing cathode of the microbial fuel cell operating on activated sewage sludge and supplemented with acetate as the carbon energy source. The maximum power density up to 1.3 Wm^−2^ (54 Wm^−3^) obtained with iron aminoantipyrine (Fe-AAPyr) catalyst is the highest reported in this type of MFC and shows stability and improvement in long term operation when continuously operated on wastewater. It also investigates the ability of this catalyst to facilitate water extraction from the anode and electroosmotic production of clean catholyte. The electrochemical kinetic extraction of catholyte in the cathode chamber shows correlation with power performance and produces a newly synthesised solution with a high pH > 13, suggesting caustic content. This shows an active electrolytic treatment of wastewater by active ionic and pH splitting in an electricity producing MFC.

## Highlights

• Long-term investigation into Fe-AAPyr catalyst in Microbial Fuel Cell.
• The maximum power density up to 1.3 Wm^−2^ (54 Wm^−3^).
• Stability and improvement in time (1 year) when continuously operated on wastewater.
• Electrochemical extraction of catholyte shows correlation with power performance.
• Catholyte solution (pH > 13) prevents biofouling and could be used as disinfectant.

## Graphical abstract

**Figure:**
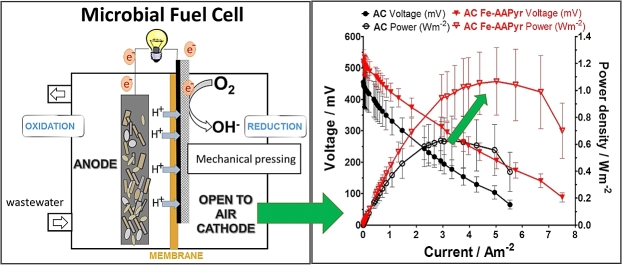

## 1 Introduction

One of the most important challenges that the world is facing today is inadequate access to clean water and sanitation. With water scarcity occurring globally, even in regions currently considered water-rich, new methods of purifying water at lower cost and with less energy, with minimal use of chemicals and impact on the natural environment, are needed. Recovery of energy and nutrients locked in municipal wastewater is one of the most promising sustainable options for organic waste reuse. Science and technology needs to be developed to improve the disinfection and decontamination of water [1]. This could be achieved with the Microbial Fuel Cell technology, which could help address the challenge of sustainability [2] and provide energy recovery [3]. In recent years, this technology has been proven to generate electricity from a variety of substrates [4], [5] including wastewater and human urine [6] and shown to have the potential for direct electricity usage to power practical applications such as robotic systems [7], mobile phones [8], [9] and indoor lighting in remote areas as presented in recent field trials [10].

Oxygen reduction electrocatalysis is of great importance for many energy storage and conversion technologies, including fuel cells, batteries and water electrolysis [11]. Replacing noble metal-based electrocatalysts with highly efficient and inexpensive noble-free oxygen electrocatalysts is critical for the practical application of these technologies [12], [13]. Cathodic oxygen reduction reaction (ORR) requires the development of effective electrocatalysts to facilitate power output in MFCs. Platinum is expensive, rare and prone to catalyst poisoning [14], [15], [16], especially when used in activated sludge environments [17], which limit the applicability of this technology and diminishes the long-term performance of MFCs. The development of precious metal-free catalysts [18], [19] such as iron rich nanoparticles on porous nitrogen-doped carbon material [20] or iron and nitrogen functionalised graphene [21] has been recently pursued. A platinum group metal-free (PGM-free) electrocatalyst consisting of M-N-C network (where M = Mn, Fe, Co, Ni, N = nitrogen and C = carbon) has been widely used in conventional fuel cells [22]. One of the aforementioned catalysts is a PGM-free catalyst material (Fe-AAPyr) synthetized using the Sacrificial Support Method (SSM) [23], [24], [25]. Fe-AAPyr derived from the pyrolysis of iron salts, aminoantipyrine as C-N precursor (Fe-AAPyr) on monodispersed silica as template, has demonstrated better performance than platinum, cobalt, nickel and manganese in single chamber MFCs under “clean” conditions (PBS) in neutral pH [26]. It showed almost no degradation in performance and high resistance against organic pollutants and anions [27]. The carbonaceous materials such as activated carbon (AC) obtained from various sources [28] is another cost effective alternative used in many MFC studies [17], [29], [30], [31]. Activated carbon has been modified using carbon black [32], stainless steel [33], metal oxides [34] and carbon fibre veil [35]. Also, Fe-AAPyr addition to activated carbon showed significant improvement in power performance as reported recently [36]. Moreover, catalyst poisoning tests demonstrated that activity decreases only slightly after immersion in sulphide and sulphate solutions [27]. While the electrochemical characterisation is clearly suggesting the suitability of PGM-free materials as catalysts for ORR [37], [38], this still requires to be tested in an operating MFC system with real wastewater for longterm evaluation to check its suitability for real world implementation and market readiness. This study looks into the integration of a Fe-N-C catalyst with a carbon-based material such as activated carbon, since AC cathodes in a similar design of MFC outperformed a range of other carbonaceous materials and it is often used in literature as control material [39], [40]. This work is a long-term investigation of the aforementioned Fe-based catalyst in the MFC cathode and demonstrates power generation over 1 year of continuous operation under wastewater feeding conditions. The study also investigates the suitability of the Fe-N-C based catalyst to produce a highly alkaline catholyte for carbon capture as the direct result of electricity production in MFC systems.

## 2 Materials and methods

### 2.1 Electrode preparation

Air-cathodes used for this study measured 10 cm^2^ were made of two types of materials: carbon veil (20 g m^−2^, PRF Composites, UK) and carbon cloth (Fuel Cell Earth, USA). Carbon veil was used as control (CV) and coated with conductive paint (CV P) (Timcal Li-quid 101, Switzerland).

Fe-AAPyr was prepared by wet mixing of iron salt (iron nitrate) and aminoantipyrine precursor with the high surface area fumed silica (EH-5) as previously described [38]. Particularly, the resultant mixture was ultrasonically treated and dried on air at 85 °C overnight. The solid composite was then ground until a fine powder was formed. After this step, heat treatment was performed in UHP (Ultra High Purity) nitrogen (flow rate 100 mLmin^−1^), T = 950 °C, t = 45 m and a temperature ramp of 25 °Cmin^−1^. After heat treatment, silica was removed using 20 wt% HF and then the obtained material was washed and neutralised using deionised water.

Two sets of cathodes were tested and their performance was compared. In one of the sets, carbon veil (CV) was used as gas diffusion layer and current collector. CV was also modified adding conductive acrylic paint (PA, Liquid 101, Timcal, Switzerland). Finally, Fe-AAPyr was added on carbon veil+ conductive paint through a dropcast technique with a loading of 0.3 mgcm^−2^. In parallel, carbon cloth (CC) was employed as an alternative gas diffusion layer and current collector with pressed activated carbon (Norit SX Plus, Sigma Aldrich) and PTFE (8:2 ratio) was run and tested. AC was used as active layer due to its intrinsic high surface area that enhances ORR. Fe-AAPyr was also added on CC + AC via a spraying technique with catalyst loading of 2 ± 0.2 mgcm^−2^. All tested cathode materials are shown in Table 1.

**Table 1:** Cathode tested during this investigation.

Acronym	Cathode	Gas diffusion layer	Active layer
CV	Carbon veil	Carbon veil	–
CV P	Carbon veil coated with conductive paint	Carbon veil	Conductive paint
CV P Fe-AAPyr	Carbon veil coated with conductive paint and Fe-AAPyr	Carbon veil	Conductive paint + Fe-AAPyr
AC	Activated carbon	Carbon cloth	Activated carbon
AC Fe-AAPyr	Activated carbon + Fe-AAPyr	Carbon cloth	Activated carbon + Fe-AAPyr

### 2.2 MFC reactor construction and operation

**Fig. 1:**
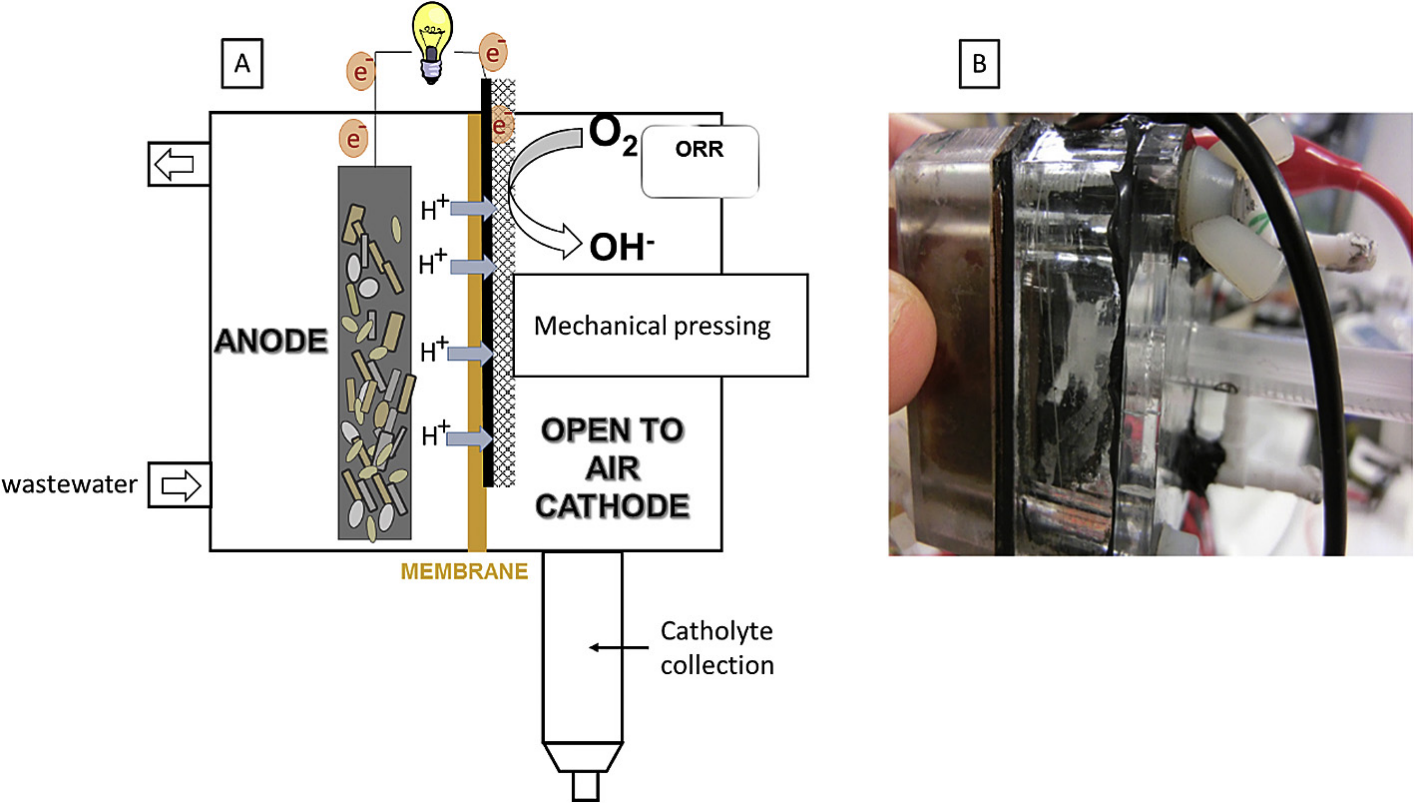
Microbial Fuel Cell scheme (A) and the photograph of single MFC unit (B).

The MFC anode and cathode chambers were constructed using transparent acrylic chambers with a total volume of 25 mL each as represented in Fig. 1. Anode electrode was set up using carbon veil fibre (20 gm^−2^, PRF Composites, UK) of a total macro surface area of 270 cm^2^ which was further folded and tied with a Ni-Cr wire for current collection. The anode chamber was connected via an inlet and outlet to the 1 L recirculation tank using silicon tubing and 16-channel peristaltic pump (Watson Marlow, UK) to maintain stable feeding conditions at a constant flow rate 48 mLh^−1^. The MFC anodes were pre-established in long term MFC conditions (over 1 year operation) and during the start-up they were inoculated with anaerobic sewage sludge (Wessex Water, UK) at the neutral pH. Anolyte used in this experiment was a mixture of activated sewage sludge supplemented with 0.1 M sodium acetate as carbon source in the 1 L feedstock tank and recirculated through the MFCs back to the reservoir. The membrane used in this study was a cation exchange membrane CMI 7000 (Membranes International, USA). Air-breathing cathodes as described in Table 1 were placed in empty 25 mL cathode chambers to maintain sufficient moisture levels and to protect the cathode from excessive evaporation. The cathode electrode (10 cm^2^) was pressed mechanically (active side facing the membrane, gas diffusion layer facing the air side) to maintain good contact with the membrane as shown in Fig. 1B. A 10 mL syringe was attached at the bottom of each cathode chamber with a use of non-toxic sealant (Wet Water Sticky Stuff, 123Aquatics, UK) to allow catholyte collection (Fig. 1A). All MFCs were tested in triplicates. The MFC performance was investigated over a period of 350 days (∼12 months).

### 2.3 Data acquisition and polarisation procedures

The MFC output voltage was measured at regular time intervals using the ADC-24 (Pico Laboratories, UK) data logger connected to a PC. Current I in Amperes (A) was derived by dividing the recorded voltage by the known external resistance value and power output P in Watts (W) was calculated by taking the product of voltage and current, i.e. P = I × V and power density (Wm^−2^) and current density (Am^−2^) were obtained by dividing power and current by cathode surface area (m^2^), respectively taking into account the cathode projected surface area of 10 cm^2^ (cathode surface inside the MFC in the direct contact with the membrane). Volumetric power and current density were calculated taking a total anodic volume (25 mL). Bioelectrochemical behaviour of MFC was assessed by performing polarisation experiments using an automated polarisation equipment [41] that applied a resistance in the range of 30 kΩ to 11 Ω in 3 min intervals between.

## 3 Results and discussion

### 3.1 Catalyst on carbon veil substratum

**Fig. 2:**
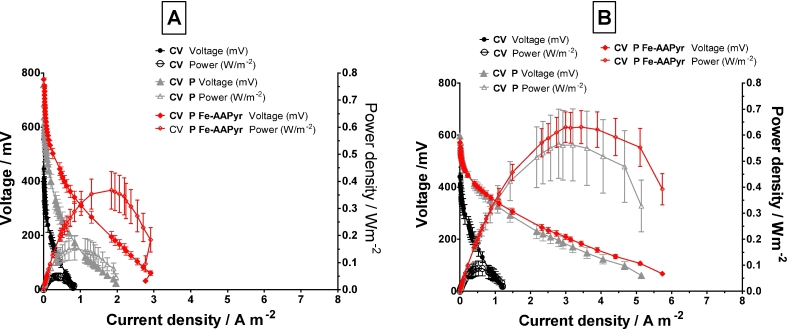
Polarisation and power curves performed on all tested materials at the beginning of the experiment (A) and after 100 days of operation in MFCs (B). Data represents triplicate MFC units with SD error bars.

Fig. 2A shows that in terms of the carbon veil substratum, the applied layer of the catalyst was significantly improving the MFC performance from 0.05 Wm^−2^ to 0.37 Wm^−2^ (mean values) which is greater than 7-fold increase. Fig. 2B illustrates the performance recorded after 100 days of continuous MFC operation where a significant improvement can be seen in all three types of cathodes. In terms of the polarisation experiment performed after long term operation, the control cathode made out of carbon veil (CV), the application of the conductive carbon paint (CV P) as well as the catalyst (CV P Fe-AAPyr) shows the improvement from 0.09 Wm^−2^ to 0.56 Wm^−2^ and 0.63 Wm^−2^ (mean values), respectively. This might be due to the better acclimation period, catholyte formation and the maturing of the anodes. Moreover, as the MFCs were operated in recirculation mode, the more power MFCs were producing, the more catholyte formation could be observed which will be discussed later in the text. The catholyte was formed on the surface of the cathode electrode dripping into the collection vessel (syringe) as shown in Fig. 1A. This allowed better hydration of the cathode without the immediate flooding and led to further performance increase suggesting that it indeed does not limit the oxygen diffusion into the active layer of the cathode but instead, it might improve ionic transfer. Higher levels of hydration of the membrane-cathode interface would allow an improved flow of ionic exchange resulting in higher power levels.

### 3.2 Catalyst on activated carbon substratum

**Fig. 3:**
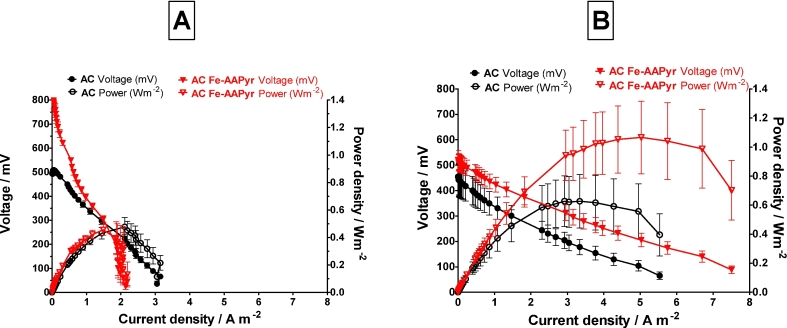
Polarisation and power curves performed on activated carbon (AC) based materials at the beginning of the experiment (A) and after 100 days operation (B). Data represents triplicate MFC units with SD error bars.

Fig. 3A shows polarisation curve experiment performed at the start of the test where both AC and AC + Fe-AAPyr performed similar showing 0.48 Wm^−^^2^ and 0.46 Wm^−2^ respectively. It is interesting that the open circuit voltage of the AC + Fe-AAPyr is significantly higher reaching up to 921 mV in comparison to AC which is showing only 514 mV. The initial contribution of the catalyst is not as visible as in the CV type of cathode in Fig. 2A and this might be due to the different nature of the fibrous carbon veil and microporous activated carbon which would have affected the coating. This initial performance of the AC is however almost 10 fold better than the control carbon veil (Fig. 2A) and slightly better than initial carbon veil with applied catalyst. This shows that activated carbon applied on carbon cloth is a preferable material to build the cathode electrode rather than using carbon veil. This is probably due to the nature of the substratum (carbon cloth and carbon veil) but also on how AC would be introduced on each material. It might also be due to the fact that carbon veil is a thinner and more brittle material used as a gas diffusion layer than carbon cloth. Again, after 100 days of operation the performance increased and in terms of the AC Fe-AAPyr it reached up to 1.06 Wm^−2^ (mean) and 1.35 Wm^−2^ (maximum) whereas for the AC the mean value was 0.63 Wm^−2^. One of the replicates reached up to 1.35 Wm^−2^ maximum recorded power which is the highest power output recorded in this type of MFC in comparison to previous studies [30], [42]. In terms of the volumetric power density it corresponds to: 43 Wm^−3^ (mean value) and 54 Wm^−3^ (maximum) for AC Fe-AAPyr and 25 Wm^−3^ (mean value) and 33 Wm^−3^ (maximum) for AC cathode. In both cases carbon veil and carbon cloth serves mostly as a gas diffusion layer and current collector. Activated carbon used here as the active catalytic cathode shows promising results, which remain in agreement with previously reported work [30], [42], however the highest power output was recorded when the Fe-based catalyst was applied onto the activated carbon cathode. This is in agreement with earlier studies investigating the catalyst using electrochemical methods in the half-cell [36]. Carbon cloth used as the gas diffusion layer is more rigid and with higher carbon loading therefore it outperforms the carbon fibre veil. This might be due to the fact that it will behave as a better conductor and has a better contact with the membrane. However, as indicated by previous work, CV was the most cost effective support when modified with conductive paint as high cost of CC does not seem suitable for large-scale applications [36] therefore AC paste could be applied to other GDL substrates such as hydrophobic carbon veil which proven good performance [35]. The carbon veil coated with activated carbon already has also been successfully used in ceramic based MFCs [35], [43] and the future study will include the incorporation of the catalyst into this configuration.

### 3.3 Long term performance

**Fig. 4:**
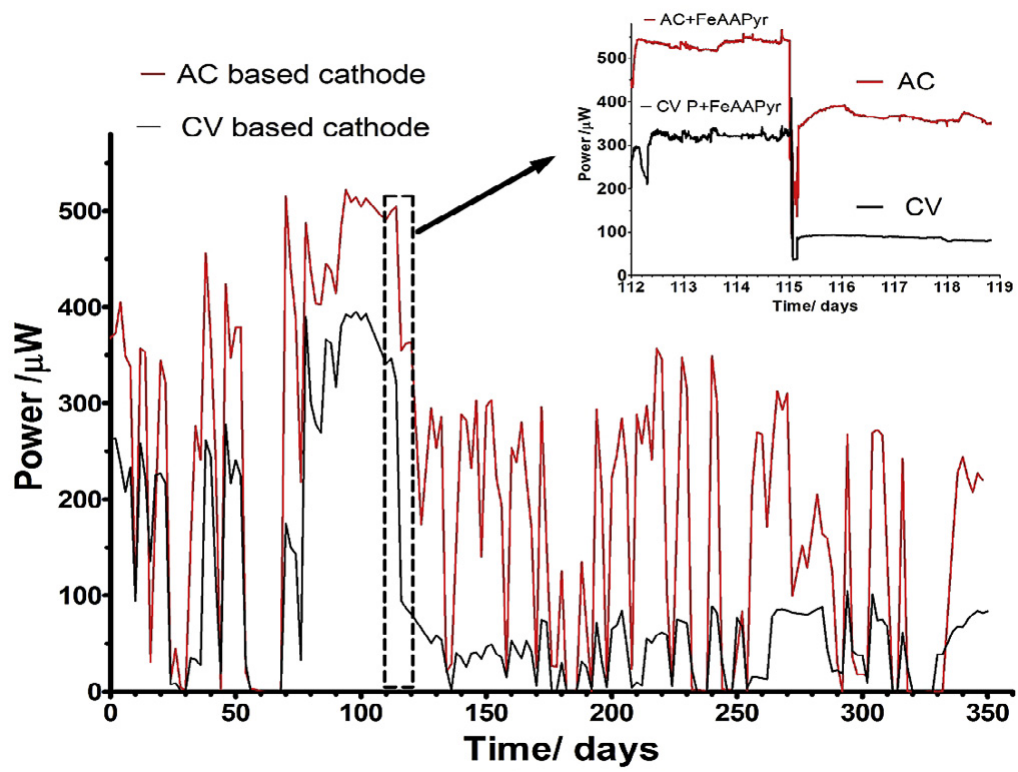
Temporal power behaviour of the MFCs, over 350 days of operation. The MFCs (mean values of triplicated data) were fed with activated sludge wastewater supplemented with sodium acetate as the carbon energy source. The arrow indicates the cathode replacement from catalyst based to non-catalyst based material (inset).

As previously discussed, the application of the catalyst onto the cathode in running MFC and long term experiments are rarely reported and up to our best knowledge only in one case Fe-N-C ORR catalysts was used for 18 months [44]. This test was maintained for the period of 12 months to observe the ability of the MFC to improve in time and to study the stability of the catalyst in wastewater operated system. The periodic feeding was at times interrupted allowing for periods of starvation, and further supply of fresh feedstock allowed performance recovery (Fig. 4). Platinum catalysts are normally prone to contamination and poisoning within the first week of operation [14] which is not the case here with the use of the Fe-AAPyr catalyst.

When the Fe-based cathode was removed from the cathode and directly replaced with the non-catalytic (without Fe-based catalyst) control (in this case the activated carbon and carbon veil), a significant decrease in performance was observed (Fig. 4, inset). The catalyst layer in both cases showed a significant improvement even after 20 days of starvation, and the addition of fresh feedstock immediately restored the power performance. In the next 50 days, power continued to gradually improve reaching up to 550 μW (0.55 Wm^−2^) and 350 μW (0.35 Wm^−2^) under 300 Ω. It should be noted that in this case, this was not the optimum resistor value but one that was purposefully kept connected to observe the MFC performance under constant and stable conditions. During the test, both catalyst (120 days of operation) and control showed no deterioration in performance therefore cathode poisoning is being prevented. It might be due to the catholyte formation which will be discussed further in the text.

### 3.4 Catholyte analysis

**Fig. 5:**
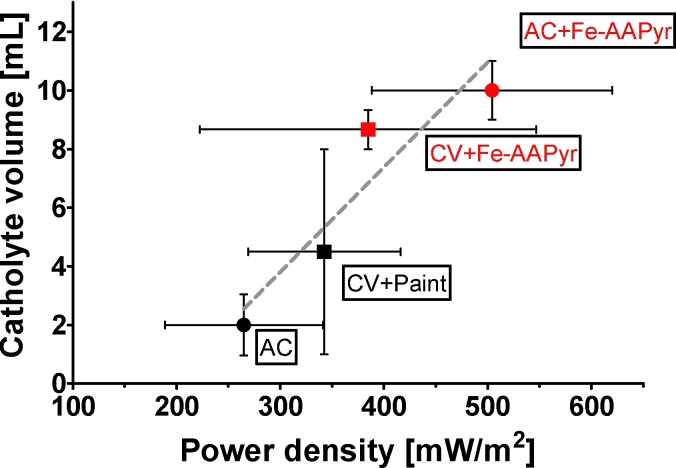
Power density during the MFC operation in relation to the collected catholyte (triplicate MFC units with SD error bars). The dotted line represents a linear regression fit that shows the direct relationship between these two parameters.

While the MFCs were monitored in terms of their electrical performance, the droplets of clear catholyte were forming on the surface of the cathodes in air-breathing set-ups, as previously described [30], [42], [43]. Formed catholyte was collected by dripping into the attached syringe (Fig. 1A). The quantity of the catholyte was correlated with the power performance as shown in Fig. 5 and it corresponds to the electroosmotic drag where water molecules are being transported, (dragged) by the cations moving in the system from the anode to the cathode [42].

**Fig. 6:**
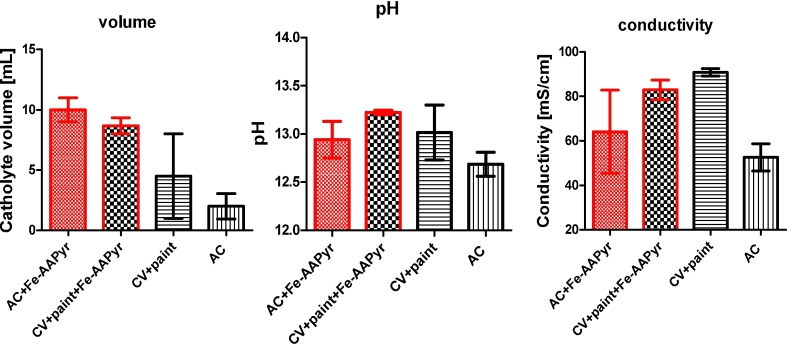
Analysis of the collected catholyte in terms of the volume, pH and conductivity values (SD error bars).

The synthesised catholyte collected in the attached syringes was transparent in colour and showed pH values of >12.5 (Fig. 6). Catholyte formation using MFCs of similar design has been presented previously [30] showing the potential for carbon sequestration and nutrient recovery [35] and offers a cost-effective technology that can selectively remove sodium [30] and potassium [42] from wastewater. Catholyte formation and nutrient recovery makes the MFC a trigenerative system in which: i) organics are degraded; ii) electricity is generated; and iii) useful chemicals are produced. Neutral pH in combination with low buffer capacities and low ionic concentrations affect the cathode performance and limit the output leading to the creation of a pH gradient and cathode alkalisation that could be actively harvested [38], [42]. In previous work, pH levels influenced the performance of both Pt and the PGM-free materials where the output decreased for the Pt and increased for the non-PGM material as the pH value increased from 6 to 11 [38]. In this work due to the formation of a catholyte that dripped into the collection vessel (away from the electrode) it is hypothesised that the pH also did not hinder the ORR. Moreover, high pH would enhance mineral sequestration of CO_2_ that requires alkalinity to form carbonate ion (CO_3_^2−^) from the CO_2_ gas. Environmental biotechnologies such as biological nitrogen removal and anaerobic digestion, where degradation of organic carbon by denitrifiers and methanogenic bacteria results in a pH increase, stimulates carbonation and carbonate precipitation [45]. However, the electrochemically formed catholyte driven via natural microbial consortia in the anode not only actively take part in carbon sequestration, but also prevent biofouling (poisoning) of the cathode. Caustic catholyte shows antimicrobial properties preventing cathodic biofilm formation and could be used as disinfectant [43]. It is assumed that the improvement in power performance and stability (Fig. 4) was due to the cathode electrode configuration that is promoting catholyte formation washing the salt deposits off and preventing biofouling [30], [42]. Cathode half-cell was designed by enclosing the air-breathing cathode in the acrylic chamber in order to prevent the cathode from drying out so that the formation of catholyte could be observed and quantified. Possibly improved MFC performance is also due to the improved water transport and better hydration of the cathode as shown in Fig. 5. Usually, the distribution of Ca^2+^, Na^+^, K^+^, phosphorous and sulphur anions, as well as biofilm growth on the cathode of single chamber MFCs affects their performance in the long-run with raw wastewater and acetate. Salt precipitation might be hindering long-term performance [46], however here, salt deposits were not observed on the cathode surface. It might be due to the design and the sufficient moisture levels as the droplets were washing down the deposits away preventing mineralisation on the cathode surface. It gives valuable grounds for further studies to focus on direct utilisation of different types of wastewater such as urine with the use of the proposed system to produce alkaline agents that help remove ammonia from the waste stream; clearly the catholyte is dependent on the nature and composition of the anolyte, as previously was shown [10], [47]. Moreover, it could lead into the electrochemical treatment of wastewater and production of disinfectant [43] as urine becomes a source of recovered electricity [10], [48] in MFC systems as well as hydrogen through urea electrolysis where urea is electrochemically oxidized in alkaline media within an electrochemical cell [49]. While the cathode scaling still limits the technology [50], the novelty of this cathode configuration in long term operating MFCs brings the development of MFC systems closer to practical demonstrations and allows to implement the technology in real life applications.

## 4 Conclusions

The main objective of this study was to investigate the platinum -free ORR catalyst such as iron based aminoantipyrine (Fe-AAPyr) in the cathode of the microbial fuel cell (MFC) and to look into the long term behaviour in real wastewater environments. A Fe-AAPyr was applied on two types of gas-diffusion cathode materials: carbon veil and activated carbon. Improvement in time was observed in both catalyst-based materials and the highest power was recorded from the catalyst applied onto the activated carbon cathode reaching up to 1.35 Wm^−2^ (54 Wm^−3^). During the 1 year of MFC operation, the efficient formation of alkaline catholyte was monitored and quantified showing the potential for extraction of valuable compounds from waste and the potential use for disinfection. In a circular economy, waste materials and energy need to be redefined as energy sources and conversion systems need to be optimised, which are elements that bring the MFC technology closer to real world applications. This is the first time that Fe-N-C type of catalysts prepared by the Sacrificial Support Method, are evaluated in long term continuous operation, which has demonstrated unprecedented activity and durability in microbial fuel cells.
